# Cold-season disasters on the Eurasian steppes: Climate-driven or man-made

**DOI:** 10.1038/s41598-018-33046-1

**Published:** 2018-10-03

**Authors:** Banzragch Nandintsetseg, Masato Shinoda, Chunling Du, Erdenebadrakh Munkhjargal

**Affiliations:** 1 Graduate School of Environmental Studies, Nagoya University, Nagoya, 464-8601 Japan; 2 Information and Research Institute of Meteorology, Hydrology, and Environment, Ulaanbaatar, 15160 Mongolia; 3 School of Arts and Sciences, National University of Mongolia, Ulaanbaatar, 210646 Mongolia; 4 School of Economics and Management, Inner Mongolia Agricultural University, Hohhot, China

## Abstract

Socio-ecological damage from climate-related disasters has increased worldwide, including a type of cold-season disaster (*dzud*) that is unique to the Eurasian steppes, notably Mongolia. During 2000–2014, *dzuds* killed approximately 30 million livestock and impacted the Mongolian socio-economy. The contributions of both natural and social processes to livestock mortality were not previously considered across Mongolia. Here, we consider the contribution of both multiple climate hazards (drought, cold temperatures and snow), and socioeconomic vulnerability (herders’ livestock and coping-capacity) to mortality risk. We performed multi-regression analyses for each province using meteorological, livestock and socioeconomic datasets. Our results show that 93.5% of mortality within Mongolia was caused by a combination of multi-hazards (47.3%) and vulnerability (46.2%), suggesting *dzuds* were both climate- and man-made. However, in high-mortality hotspots, mortality was primarily caused by multi-hazards (drought-induced pasture deficiency and deep-snow). Livestock overpopulation and a lack of coping capacities that caused inadequate preparedness (e.g., hay/forage) were the main vulnerability factors. Frequent and severe multi-hazards greatly increased the mortality risk, while increased vulnerability caused by socioeconomic changes in Mongolia since the 1990s tended to amplify the effects of multi-hazards. Thus, reductions in herder vulnerability within high-mortality hotspots would likely be an effective means of mitigating the risk of future *dzuds*.

## Introduction

Climate-related disasters, along with the associated damage to livelihoods and socio-ecological systems, have increased worldwide in recent decades^1,2^. Such disasters are especially prevalent in countries along the Eurasian steppes, such as Mongolia^3,4^. Many factors influence this increased risk of climate disaster, as disasters depend not only on climate hazards (*hereinafter* hazards) but also on the vulnerability of human communities^1,5^. A rigorous understanding of disaster risk, its causal dimensions and changing trends in those dimensions^2^, may act as a foundation for reducing future risk.

Many human societies vulnerable to hazards are also under pressure from economic and socio-political change^6^. Herders are especially vulnerable to hazards as they generally live in marginal lands^7^, such as grasslands. In Eurasian grasslands, including the Mongolian Plateau, herding and farming constitute 35% of the workforce, and occur in harsh cold and arid environments^3,4^. Because of the harsh climate, weak livestock often die during cold-season (October–March/April); however, abnormally high mortalities occur during anomalously harsh cold-season (*dzuds* in Mongolian). *Dzuds* occur throughout central Asia^3,4,7,8^, including Mongolia, Kazakhstan, Inner Mongolia and Tibet. In the 2000s, an increase in the frequency and severity of *dzuds*, together with warming^9^ and drying^10^ trends, caused massive livestock mortalities in Mongolia^3,11,12,13^. This had a large impact on the national socio-economy^11^. A single *dzud* in 2010 caused economic losses of 345 million US$^14^, and 10 million livestock mortalities (23.4% of the total livestock), which was the worst *dzud* disaster since 1945^3,11,14^.

*Dzuds* are defined, biogeophysically, as anomalous climatic and/or land surface conditions (i.e., snow and ice cover) that lead to reduced accessibility and/or availability of pastures. This causes very high livestock mortality (*hereinafter* mortality) during the cold-season^3,4,14^. Hazard-oriented studies have shown that years with high mortality result from a combination of growing-season drought and severe weather^3,12,13^. However, human-induced vulnerability^3,15–22^, including inadequate pasture management, lack of herder experience, poverty, and insufficient winter preparedness, has contributed to recent *dzuds*. This vulnerability is due in part to the transition to democracy and a free market economy in Mongolia since the 1990s^18,19,22^. Following this, formal pasture management institutions were weakened as herding collectives were dissolved^17–19^. Additionally, state structures for managing natural disasters (e.g., *dzuds*) were also weakened. During de-collectivization, state-owned livestock were privatized, state-provided disaster reduction services were stopped, and the burden of risk was transferred to herder-households^16,20–22^. This directly and indirectly increased herder-households’ vulnerability to climate extremes^3,10^. Consequently, studies of *dzud* risk must incorporate both natural and human factors.

This study attempts to fill the gap in the literature, and presents the first, to our knowledge, assessment of livestock mortality risk during the 2000‒2014 cold-season (October–March). We do this by examining both climate hazards and herder vulnerability at the Mongolian national level.

**Figure 1:**
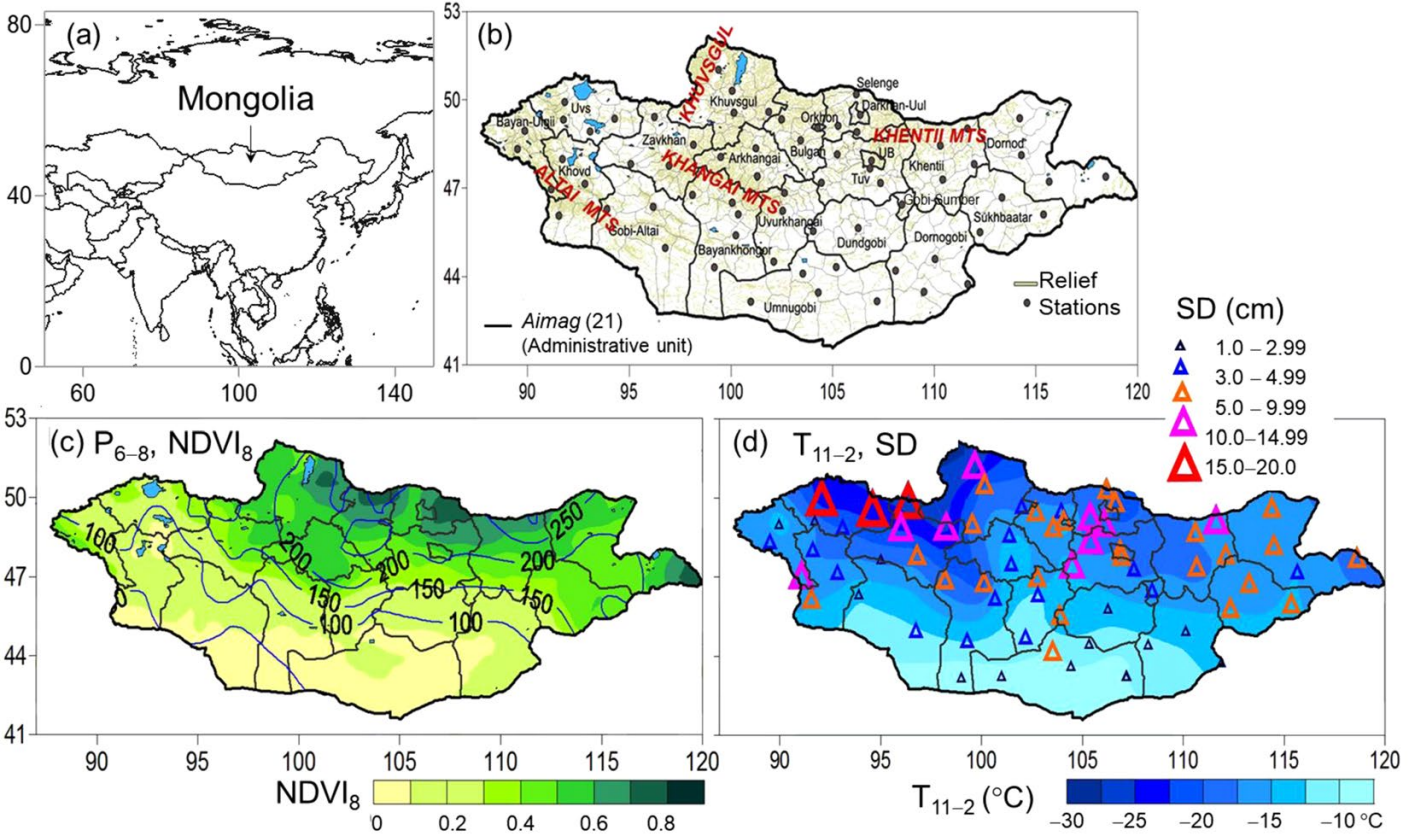
**(a)** Location of Mongolia in Eurasia; (**b**) sixty-nine station locations with province (*aimag* names) boundaries and relief lines; (**c**) climatology (1981−2014), showing precipitation (P_6−8_, mm in blue contours) for critical growing months (June–August), and peak pasture-vegetation conditions for August (NDVI_8_; background green); and (**d**) winter air temperature (background blue) in November−February (T_11−2_) and maximum snow-depth in January (SD_,_ coloured triangles).

Mongolia is located over in mid-latitude highlands in the far eastern continent and has a cold, arid climate^4,23^ (Fig. 1a,b). The latitudinal climate pattern is a key factor driving pasture production^10^, which is the only source of livestock hay and forage. Favourable conditions exist in the north and northeast, with increasingly dry conditions in the west and south. The region has long and extremely cold winters, with temperatures reaching below −30.0 °C. The air temperature remains below freezing (below 0 °C) from November through March. Pastureland is covered with snow during mid-October−April, with higher snow in northern regions, and lower snow in the low-lying south (Fig. 1d). In the fall, herders practice *otor* (transhumance) to fatten livestock so that they are more likely to survive a harsh winter^18,24^. Moreover, herders prepare hay and forage from grasslands for livestock to feed on during the cold season when they must confront freezing temperatures (Fig. 1d). These practices are a widely used and critically important traditional strategy for surviving winter hazards^18^. During winter and spring, most plants decay and pasture vegetation is maintained as dead leaves^25^ providing livestock with fodder.

## Dzud-risk (livestock mortality) framework

**Figure 2:**
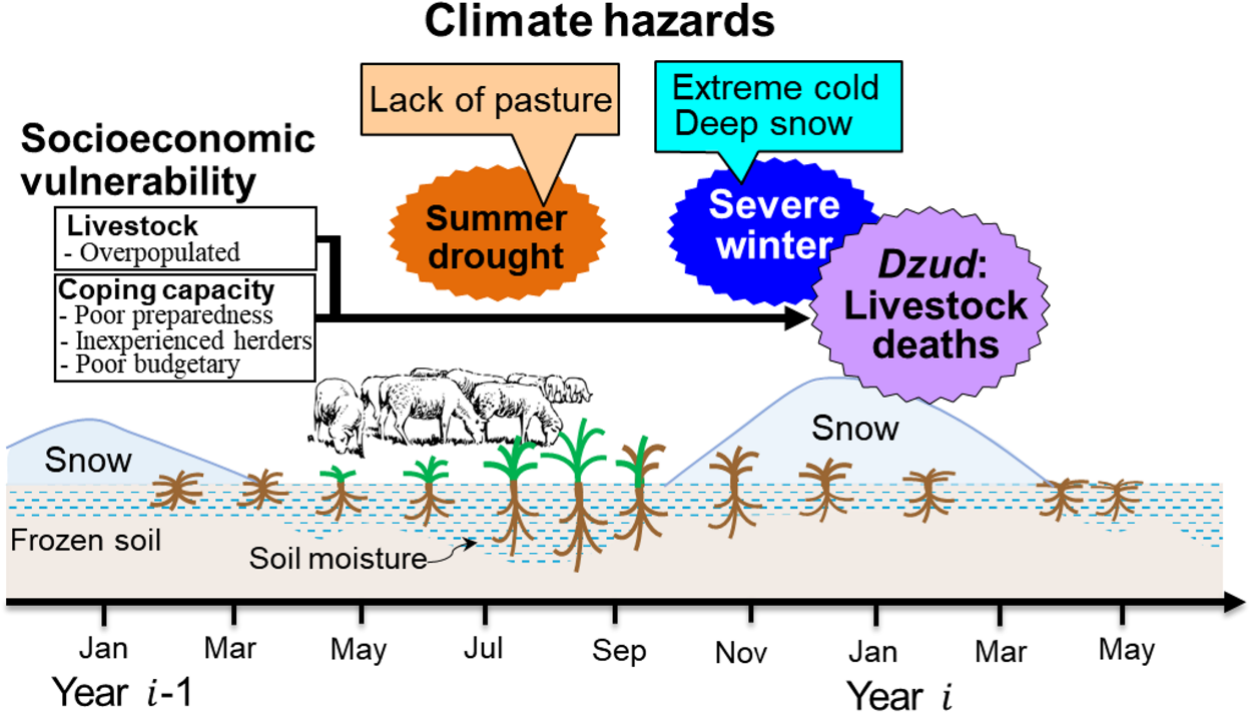
Framework of *dzud* risk (livestock mortality) that results from the combination of climate hazards (drought-induced lack of pasture in summer and autumn, and severe winters with extreme cold and deep snow) and herders’ vulnerability (pre-winter herder-households’ socioeconomic conditions). Socioeconomic conditions include weakened livestock due to small pasture capacity (overpopulated), and less coping-capacity that made herders more vulnerable to climate hazards (less availability and accessibility of pasture for livestock), thereby caused livestock to become emaciated and starve in the winter and spring.

Here, we consider the risk^5,16,26^ of livestock mortality (Fig. 2) as caused by a combination of multiple climate hazards (multi-hazards) and herders’ vulnerability, both of which may limit the availability and accessibility of pasture for livestock. The multi-hazards include drought and severe winter conditions (i.e., extreme cold and heavy snowfall). The drought declines pasture quantity and quality, preventing livestock from gaining body fat, and also prevents adequate forage preparation for cold-season feeding. Drought thus causes vulnerability for livestock and herders. In the winter, drought-induced short grasses are covered by snow that exceeds plant height, thereby preventing grazing. Extreme cold surges associated with synoptic storms reduce food intake by livestock (intake is determined by the availability of phytomass not covered with snow and ice, and grazing time). For our hazard assessment, we used anomalies of precipitation^3,12,13^ (P_6‒8_), temperature^3,12^ (T_11‒2_) and maximum snow depth (SD)^3,13^. We also used vulnerability, as defined as herder-households’ pre-winter socioeconomic ability to cope with hazards (i.e., livestock condition and coping-capacity)^17^. For the risk analysis, we selected six factors from six groups based on the highest correlations with livestock deaths (Table 1). The livestock factor included animal condition that inferred from pasture carrying capacities (PCC)^3^. High livestock population sizes (POP_pre_) reduce the pasture and forage availability for each animal, thereby increasing the likelihood of mortality^3,17^. Coping-capacity was determined by preparedness of reserve hay/forage, possession of transportation (number of cars and tractors; trucks), herder experience (fraction of herders 35‒59 years old; H_exp_), poverty (households with 101‒200 livestock; L_101−200_) and the *aimag*’s gross domestic product (GDP). The factors determining coping-capacity were related to the ability of herders to pursue *otor* (fall and winter). All these factors may combine in ways that produce very high livestock mortality, often from starvation. We used Poisson multi-regression (PMR)^27^ to determine the factors that best explained the mortality as risk indices^5^. The dependent variable was number of mortalities, and the explanatory variables included hazards (P_6–8_, SD and T_11–2_) and vulnerability (POP_pre,_ H_exp_, L_101–200_, trucks, hay/forage and GDP; see Methods).

**Table 1:** List of the selected factors of livestock mortality risk, including both climate hazards and herders’ vulnerability variables, and their averaged linear (Pearson) correlations (*r*) with livestock deaths during the 2000−2014 cold-season.

Main risk factors		Groups	Indicators		*r*
Climate hazards		Preceding summer drought	**P**_**6–8**_	Drought index (Precipitation anomaly percentage) in June−August	**−0.24**
		Severity of winter	**T**_**11–2**_	Mean temperature anomaly for November−February	**−0.35**
			**SD**	Monthly maximum snow depth for November−March	**0.41**
Herders’ socioeconomic vulnerability	Livestock conditions	Pasture capacity	**POP**_**pre**_	Previous year livestock number in sheep unit	**0.29**
		Energy conditions	LOSS_pre_	Previous year livestock mortality in sheep unit	0.04
	Coping- capacity	Herder experience	H_young_	Fraction of herders’ population aged 16‒34	0.10
			**H**_**exp**_	Fraction of herder population aged 35‒59 (males) and 35‒54 (females)	**−0.17**
		Poverty (well-being)	L_≤ 100_	Fraction of herders who have ≤100 livestock	0.02
			**L**_**101–200**_	Fraction of herders who have 201‒500 livestock	**0.16**
			L_201–500_	Fraction of herders who have 201‒500 livestock	−0.05
			L_501–999_	Fraction of herders who have 501‒999 livestock	−0.03
			L_≥1000_	Fraction of herders who have ≥1000 livestock	−0.02
		Facilities	**Trucks**	Number of cars and tractors per herder household	**−0.17**
			TV	Fraction of herders who have TVs	−0.11
		Winter preparedness	Hay/forage*	Prepared hay/fodder per livestock (sheep unit)	**−0.46**
			Shelter**	Number of warm barns and roofed barns per herder household in winter camp	−0.24
		Economy	**GDP**	Real GDP per capita (*aimag*) (10^3^ Mongolian Tugrug)	**−0.22**

Factors in bold are the indicators selected for risk analysis.

*Prepared hay/forage includes hay, imperfectly ripened wheat, artificial (concentrated) feed, salt/mineral salt and crop residuals. These are summed with coefficients as follows: 0.45 for hay, 0.35 for planted feed, 0.25 for silage feed, 1.0 for artificial feed and mineral salt, 0.22 for potatoes and vegetable scraps, 0.9 for imperfectly ripened wheat, 0.4 for residuals and 0.25 for straw. Numbers in bold are the indicators selected for risk analysis.

## Results

**Figure 3:**
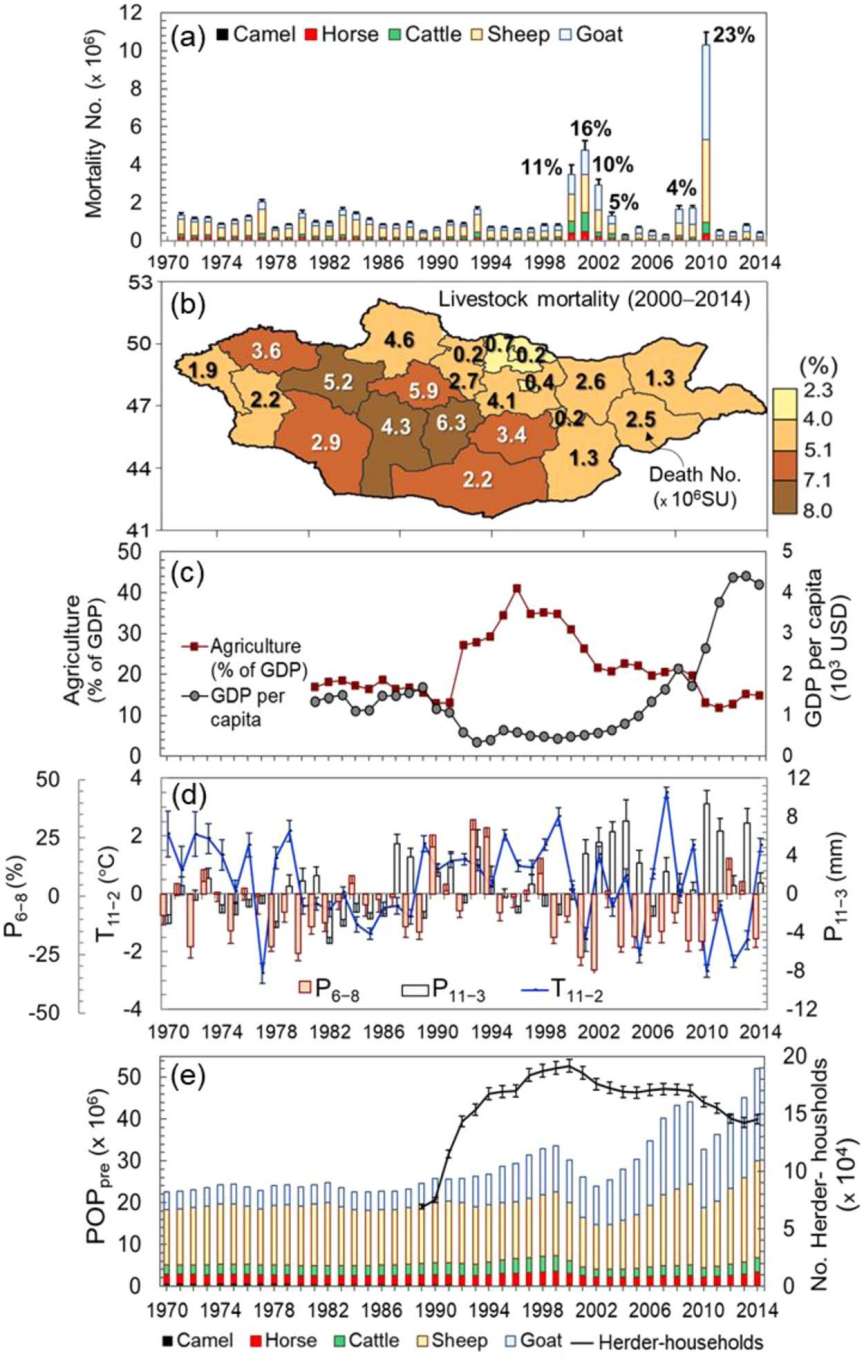
Temporal (**a**) and spatial (**b**) variation in livestock mortality, with economic damages (**c**) and major risk factors (**d**,**e**). (**b**) Spatial patterns of relative mortality (%) with their respective death numbers multiplied by 10^6^ sheep units (SU) during 2000–2014 in cold-season for each *aimag*. (**d**) Climate hazards (drought (P_6−8_), winter temperature anomaly (T_11−2_) and snowfall (P_11−3_), averaged over 69 stations). (**e**) Herders’ vulnerability (livestock POP_pre_ and number of herder-households. (**c**) Mongolian economic conditions (GDP per capita, current USD) and agricultural (agriculture, forestry, and fishing) value added (% of GDP) based on constant local currency. Error bars are regional (22 *aimags*) standard deviations (**a**,**e**) and standard errors (**d**).

Figure 3 depicts the interannual and spatial patterns of mortality, and its causal factors in Mongolia. In the 2000s (Fig. 3a), the country experienced frequent mass mortalities (a total of 30.2 million herds died, which equals 80 million sheep units, SU), with most severe mortality occuring in 2000 through 2002 and 2010 winters. The greatest losses occurred in small herds of goat and sheep, which were the most vulnerable because they were the least mobile and least able to find pasture in deep snow^22^. *Dzuds* can be large enough that they impact the Mongolian national economy, with GDP growth rates falling in response to severe *dzuds* (Fig. 3c). Regions extending from western to south-central Mongolia were high-mortality hotspots (80% of the total mortality), whereas the north-eastern and eastern regions were less affected (Fig. 3b). This regional difference may have been caused by the relative contributions of multi-hazards and vulnerability in each region.

In the 2000s (Fig. 3d), frequent climate hazards such as droughts, and severe winters with below normal cold and above normal snow have increased. Droughts caused reduced pasture biomass^10^, tending to decrease the amount and nutrition of pastures and hay/forage. Furthermore, since the 1990s, changes at the national level to the Mongolian socioeconomic system^20^ has significantly impacted herders’ vulnerability in conjunction with increased larger-scale herders (lacking herding experience) (Fig. 3e). Inexperienced herders tended to have small herds of sheep and goats, thereby changing the overall herd composition in Mongolia (Fig. 3e). These small herds were heavily affected during *dzuds*. As a result, the POP_pre_ rapidly increased, doubling doubling from 25 million heads in 1990 to 52 million in 2014 (Fig. 3e). Winter preparedness was likely also affected by less experienced herders such as declining hay/forage preparation (not shown) that reduced the availability of winter feed for large-scale POP_pre_. The lack of winter preparedness therefore likely increased the number of vulnerable herders.

**Figure 4:**
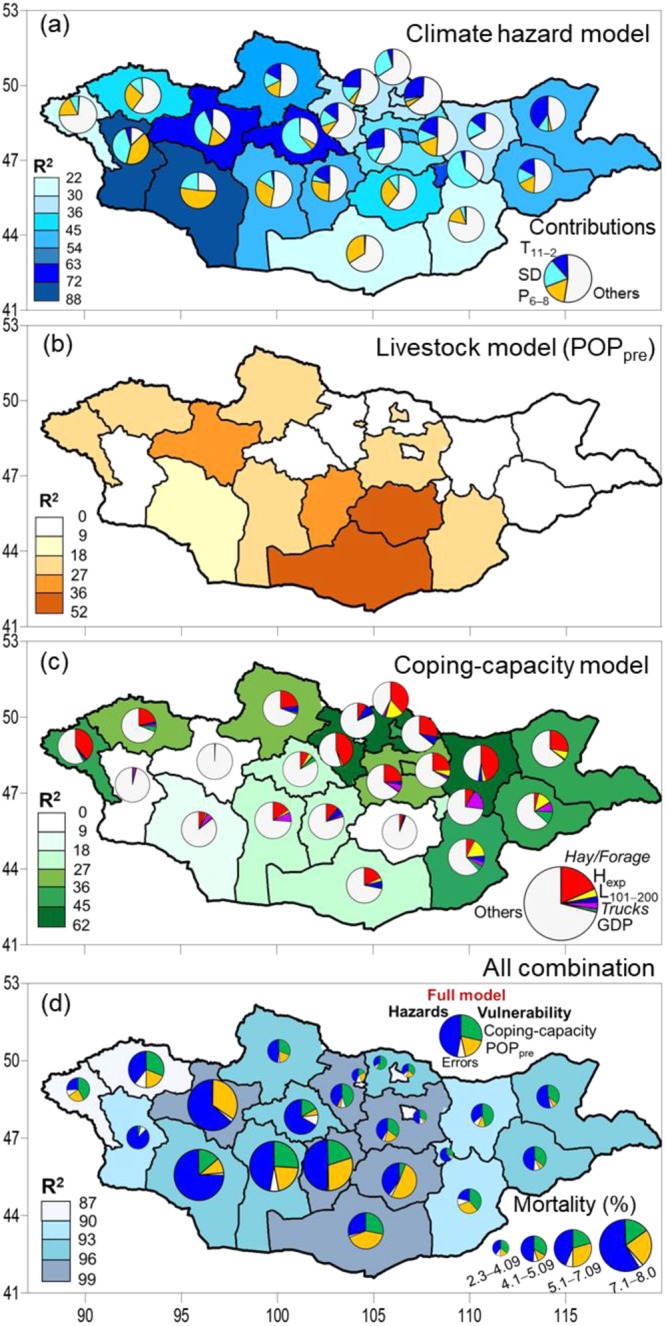
Contributions (%) of (**a**) climate hazards, (**b**,**c**) herders’ vulnerability (livestock population POP_pre_ and coping-capacity), and (**d**) their combination to livestock mortality in 2000‒2014 cold-season by cumulative variance from each model (background colours) for each aimag by PMR. Relative contributions of each factor (increment variance) to mortality showed in colour pies: (**a**) hazards of drought (P_6–8_), cold-temperature (T_11–2_) and snow (SD); (**b**,**c**) vulnerability factors of POP_pre_ and coping-capacities of hay/forage preparedness, herder experience (H_exp_), poverty (L_101−200_), transportation facilities (trucks) and economic conditions (GDP); and (**d**) all factors. In Fig. 4d, patterns of averaged mortality (percentage; sized pies) with relative contributions of hazards and vulnerability (POP_pre_ and coping-capacity) to mortality.

Figure 4 shows cross-validated PMR results for the relative contributions of risk factors of multi-hazards (Fig. 4a) and vulnerability (Fig. 4b,c) to mortality (2000−2014) for each *aimag*. Results of the leave-one-out cross-validataion (LOO-CV) analyses for the full model show value of prediction error measure *Q*^2^ > 0.9. This suggests the full model is highly predictive. The full model (Fig. 4d) accounted for 93.5% of overall mortality in Mongolia, with 47.3% by multi-hazards and 46.2% by vulnerability, indicating both factors contributed almost equally to mortality.

For the hazards, P_6−8_, SD and T_11−2_ explained 16.7%, 19.4% and 11.1% of variance in causes of deaths (Fig. 4a), respectively. This indicates that higher mortality was caused by drought and heavy snowfall. For vulnerability, POP_pre_ (Fig. 4b) and coping-capacity (Fig. 4c) explained 17.6% and 28.7% of mortalities, respectively. For coping-capacity, hay/forage (18.3%) accounted for a relatively larger contribution to deaths than the other factors (3.5% H_exp_, 3.3% trucks, 2.1% L_101−200_ and 1.5% GDP). This suggests that herder households that prepared sufficient hay/forage were more resistant to winter climate hazards. Moreover, the 4% contribution of H_exp_ to deaths likely implies that experienced herders were able to anticipate hazards in herding and pasture management. These results indicate that POP_pre_ and prepared hay/forage were the most crucial vulnerability factors.

In high mortality hotspots (5.1–8.0% of total livestock deaths; Figs. 3b and 4d), major mortality (average of 51.1%, ranging from 29.0–74.3%) was caused by multi-hazards (26.7% by P_6−8_, 20.7% by SD and 3.4% by T_11−2_). This suggests that those regions had harsher climates (more severe drought- and snow-related livestock mortality; not shown). For vulnerability, contributions of high POP_pre_ (26.4%) and low coping-capacity (17.9%, with 10.5% by hay/forage) were found. This indicates that overpopulated livestock and higher drought risk both contributed to a lack of pasture for livestock (overgrazing), causing weakened animals and reduced hay/forage preparation. This in turn left herders more vulnerable. Moreover, the hotspots contained fewer experienced herders and trucks, as well as a lower GDP, likely limiting the ability of herders to practice *otor* and prepare hay/forage. The mortality hotspots faced severe winter hazards, with insufficient winter feed for weakened livestock because of previous overgrazing.

Conversely, low mortality regions (<5.1% of total livestock deaths; Figs 3b and 4d) generally coincided with relatively low vulnerability and higher (average of 44.4%, ranging from 25.0–63.9%) contributions of the low vulnerability factors, resulting in reduced mortality. Of the multi-hazards, winter climate hazards (17.3% from SD and 16.1% from T_11−2_) outweighed drought (10.4% from P_6−8_). For vulnerability, coping-capacity (35.3%, with 22.9% from hay/forage) was a large contributor to low mortality, while POP_pre_ had a minor influence (13.0%). Furthermore, these regions had higher proportions of H_exp_, trucks and GDP, likely facilitating the ability of herders to prepare hay/forage and practice *otor*. Low mortality regions thus had sufficient coping-capacity and PCC, as well as lower drought risks.

## Summary and Discussion

From 2000 to 2014, *dzuds* (including century-worst *dzud* events) killed approximately 30 million livestock, and greatly impacted the Mongolian socio-economy. To understand the natural and man-made causes of these *dzuds*, this study presents the first, to our knowledge, assessment of contributions of both climate hazards and socioeconomic vulnerability to livestock mortality risk. We performed PRM analyses for each province using meteorological, livestock and socioeconomic datasets. Our results show that cross-validated PMR performed reasonably well in explaining the variation in mortality (*R*_*M*_^2^ = 93.5%), with sufficient predictive ability (*Q*^2^ > 0.9). We found variations in mortality were caused by a combination of multiple climate hazards and herder vulnerability. Climate hazards, particularly droughts, have increased in Mongolia since 2000^10^, with droughts being unusual during the previous millennium^28^. In addition, recent anomalously severe winters in Mongolia have been linked to declines of Arctic sea-ice that in turn triggered changing atmospheric circulation, causing extreme winters in mid-latitude Eurasia^29,30^. These increasing multi-hazards led to pasture deficiency and a continuing loss of adequate grazing, causing significant mortality in cold-season.

Additionally, greater herder vulnerability caused by economic and socio-political changes (including weakened state-based disaster risk management) since the 1990s amplified livestock mortality levels. Livestock overpopulation and the lack of herder coping-capacity, along with inadequate hay/forage preparedness, were the main factors causing this vulnerability. Over the past two decades, traditional herding strategies were affected by increasingly inexperienced herder-households, as well as herd compositions changed to smaller animals and increased livestock population sizes^3^. Moreover, many herders have reduced or stopped practicing *otor* to avoid labour and transportation costs^24^. This herders’ vulnerability, combined with declining pasture production brought on by drought, has resulted in overgrazing^3,24^, which was the primary reason for the record mortality in the 2009/2010 *dzud*^3^. A lack of data availability prevented us from analysing other aspects of vulnerability, such as herder behaviour (e.g., *otor*) and herding environment (e.g., access to water).

High mortality hotspots in western and south-central Mongolia were caused by livestock overpopulation^3^ and large herder vulnerability^17^. The hotspots had certain geographical disadvantages of being located in arid and mountains regions. The southern region was arid with poor pastures, while high mountain regions in the western region were prone to heavy snow, hindering the ability of the state to deliver emergency forage. Another disadvantage of these regions was their remoteness, being located far from the capital, Ulaanbaatar, where market opportunities as well as disaster relief were available^17^. Conversely, regions with low mortality had sufficient PCC because they were located in relatively wet plains with favourable pasture, as well as having herders with low vulnerability. Additionally, they had the advantage of being located near major cities, ensuring effective market access^17^, and providing better infrastructure in the form of roads for delivering emergency forage.

Herding husbandry, particularly in vulnerable hotspots, likely will continue to face increasing future climate risks (such as droughts^28^ and severe winter^30,31^). As a consequence, alleviating the impacts of climate change on herder communities, through strengthening adaptive capacities, risk reduction strategies (including reducing herder vulnerability to future hazards) and resilience in degraded environments, will be a crucial challenge. Moreover, it is crucial to develop an effective early warning system (EWS) for climate hazards by improving weather forecasts and considering all factors influencing vulnerability. The *dzud* risk model presented in this study should be incorporated into a future *dzud*-EWS to include the quantitative contributions of natural and socioeconomic factors in the warning system. This approach should be extended to other Eurasian steppe regions^4^ that share the same climate change and herding challenges as Mongolia.

## Material and Methods

### Datasets

Data for livestock population sizes (POP) and mortality from hazards at the *aimag* (the administrative unit of Mongolia; there are 21 *aimags*) level from 1971 to 2014 were obtained from the National Statistical Office (NSO) of Mongolia (NSO, 2000–2014)^32–35^. The NSO conducts annual surveys in December of the total number of horses, cattle, sheep, goats and camels in Mongolia. These data were converted into sheep units (SU) to standardize feed requirements as each type of livestock requires different amounts of feed^3^. Conversion rates to SU were: 1 camel = 5 SU, 1 horse = 7 SU, 1 cow = 6 SU, 1 sheep = 1 SU and 1 goat = 0.9 SU. The percent relative mortality for each *aimag* was calculated as the ratio of the total number of livestock deaths during winter and spring to the total number of livestock at the beginning of the year.

To assess climate hazards, we used monthly average air temperature (T), precipitation (P) and maximum snow depth (SD) from 69 stations in Mongolia from 1971 to 2014 (Fig. 1b). These data were obtained from the Information and Research Institute of Meteorology, Hydrology and Environment of Mongolia. For pasture vegetation, we used a monthly 0.5° × 0.5° grid normalized difference vegetation index for August (NDVI_8_) from the semi-monthly 8-km resolution Global Inventory Modelling and Mapping Studies (GIMMS)^36^ for the period 1982–2006. We also used the 16-day 0.05° × 0.05° spatial resolution Moderate Resolution Imaging Spectroradiometer (MODIS)^36^ data from 2007−2014. (Fig. 1c). Droughts were evaluated based on the plant-available precipitation anomaly percentage during the critical growing months of June through August (P_6‒8_)^3^. The severity of winter was assessed based on anomalies of T for November–February (T_11‒2_)^3,12^, and maximum snow depth (SD) in January, which showed the strongest significant relationship with losses^3,13^. Anomalies were based on the period of 1981‒2010.

For vulnerability, we considered socioeconomic data related to pre-winter herder households available at the *aimag* level from 1999−2013. Datasets were compiled using the livestock census conducted in December by the NSO^17,32–34^. The gross domestic product (GDP) for Mongolia (per capita in current USD and value added in the agricultural sector as percentage of GDP) as an indicator of economic conditions were taken from the World Bank during 1981–2014^35^. We selected 14 indicators encompassing six groups (one group for conditions of livestock and five groups for coping-capacity; Table 1). The preceding livestock mortality (LOSS_pre_)^13^ and population size (POP_pre_)^3^ were selected as livestock factors. We measured household possession of transportation as the number of cars and tractors (trucks), possession of tools to acquire information on weather and pasture through televisions, economic conditions by *aimag*’s GDP, herders’ experience by fraction of aged herders (H_exp_) and well-being or poverty conditions by proportion of households who have 101−200 livestock (L_101−200_). The number of livestock owned is a common measure of wealth/poverty in rural areas^24^. For the risk assessment, we selected six factors based on the highest correlation with losses from each group (Table 1): POP_pre_, H_exp_, L_101−200_, trucks, hay/forage and GDP.

### Study area

Most areas of Mongolia located in more than 1500 m above sea level. Elevation ranges between 800 and 4000 m, with marked differences in elevation from west to east and north to south. Climate and topography organize the terrain into pronounced eastern, central, western and southern regions. Eastern Mongolia is composed of flat or undulating plains^23^. The central to north-western regions are made up of the Altai, Khangai, Khentii, and Khuvsgul mountain ranges (Fig. 1b), interspersed with depressions or basins. The southern region encompasses the Gobi Desert. The annual precipitation varies from over 300 mm in the north to below 100 mm in the south and is concentrated in June-August (Fig. 1c). The pasture growing season is short (May–August)^10^ and peaks at the end of August (normalized difference vegetation index, NDVI_8_; Fig. 1c). Pastureland is covered with snow from mid-October to the end of April, and the yearly maximum snow depth is observed in January, ranging from over 100 mm in the northern mountains to below 10 mm in the south (Fig. 1d).

### Poisson regression model and cross-validation

We used Poisson multi-regression (PMR)^27,37^ to determine the factors that best explained the livestock deaths as risk indices^5^. Death counts are typically distributed through a Poisson process, generating independent and random occurrences across time or space. If time were divided into discrete periods, death counts would be theoretically distributed as a Poisson distribution^27,37^. The probability distribution of the number of occurrences, *Y*, of some random event in an interval of time or space is (Eq. 1):

1 $${\rm{\Pr }}(Y={y}_{i})=\frac{{e}^{-{\mu }_{i}}{({\mu }_{i})}^{{y}_{i}}}{{y}_{i}!}\,({y}_{i}=0,1,2,3\ldots )$$

where ***y***_***i***_ is the number of deaths for a particular time ***i***, and ***μ***_***i***_ is the expected number of deaths per period. The mean and the variance of the distribution are both ***μ***_***i***_. The mean and variance of this distribution is ***y***_***i***_ ~ Pr(***μ***_***i***_). Poisson multiple regression (PMR) is a basic method for analysing the relationship between observed mortality and a set of explanatory variables. In this study, the dependent variable was total livestock deaths of five species of livestock during 2000−2014 cold-season, which were converted to SUs to standardize feed requirements. The factors selected as explanatory variables were multi-hazards (P_6–8_, SD and T_11–2_) and vulnerability (POP_pre,_ H_exp,_ hay/forage, L_101−200_, trucks and GDP). In this study, we assumed that observed livestock deaths occurred over a fixed time interval, and because the counts were nonnegative, PMR is defined in terms of log of expected deaths (Eq. 2):

2 $${\mu }_{i}=\exp ({\beta }_{0}+{\beta }_{1}{x}_{1}+{\beta }_{2}{x}_{2}+\ldots {\beta }_{i}{x}_{i})$$

where *x* is the explanatory variable and *β*_*i*_ is the regression coefficient. Thus, the typical PMR expresses the log outcome rate as a log linear function of a set of predictors. Initially, the PMR included only P_6–8_, each hazard of SD and T_11–2_. We then added vulnerability as POP_pre_ and finally added all variables, including each coping-capacity factor. Separate models were developed for each *aimag*, because the contributions of each factor to deaths vary from region to region. The contribution of a given predictor is measured by the increment of variance (*R*_*M*_^2^; McFadden’s Pseudo-*R*^2^) based on log-likelihoods that are calculated from adding each predictor to the model. *R*_M_^2^ closely parallels *R*^2^ in linear regressions, both conceptually and mathematically^38^. The measure may be thought of intuitively as a proportional reduction in error measure. To measure the relative importance of predictors, we performed a dominance analysis, which is an examination of the *R*_M_^2^ values for all possible subset models. This is performed by calculating each predictor’s added predictive ability in the presence of other predictors^38^.

To evaluate the full PRM model’s predictive ability, the leave-one-out cross-validation (LOO-CV) analysis^39^ was applied for each *aimag*. The LOO-CV is a commonly used error estimation technique, and it consists of excluding each sample once, constructing a new model without this sample, and predicting the value of its dependent variable, *y*_*i*_. In the LOO-CV, each observation in the sample dataset of size *n* (*n* = 15 years in this study) is successively taken out and the remaining *n* − 1 observations of the set are used to train the prediction model to estimate the livetsock mortality. This process is done *n* times. The predictive ability of the model is assessed by prediction error measure, *Q*^2^ (Eq. 3):

3 $${Q}^{2}=1-\frac{{\sum }_{i=1}^{n}{({\hat{y}}_{i}-{y}_{i})}^{2}}{{\sum }_{i=1}^{n}{({y}_{i}-{\bar{y}}_{i})}^{2}}\,$$

where *y*_*i*_ and ${\hat{y}}_{i}$ are the observed and predicted livetsock mortalities for an individual compound in the training set. The value ${\bar{y}}_{i}$ is the mean value of *y* for all samples. The ${Q}^{2}$ statistic provides cross-validation, as well as a qualitative measure of consistency between the predicted and original data. An acceptable value for ${Q}^{2}$ in biological models is ≥0.4^39^.
