# Author Correction: Cold-season disasters on the Eurasian steppes: Climate-driven or man-made

**DOI:** 10.1038/s41598-018-33871-4

**Published:** 2018-10-23

**Authors:** Banzragch Nandintsetseg, Masato Shinoda, Chunling Du, Erdenebadrakh Munkhjargal

**Affiliations:** 1 Graduate School of Environmental Studies, Nagoya University, Nagoya, 464-8601 Japan; 2 Information and Research Institute of Meteorology, Hydrology, and Environment, Ulaanbaatar, 15160 Mongolia; 3 School of Arts and Sciences, National University of Mongolia, Ulaanbaatar, 210646 Mongolia; 4 School of Economics and Management, Inner Mongolia Agricultural University, Hohhot, China

Correction to: *Scientific Reports* 10.1038/s41598-018-33046-1, published online 03 October 2018

This Article contains errors in the Results section.

“In the 2000s (Fig. 3a), the country experienced frequent mass mortalities (a total of 30.2 million herds died, which equals 80 million sheep units, SU), with most severe mortality occuring in 2000 through 2002 and 2010 winters.”

should read:

“In the 2000s (Fig. 3a), the country experienced frequent mass mortalities (a total of 30.2 million herds died, which equals 57.4 million sheep units, SU), with most severe mortality occurring in 2000 through 2002 and 2010 winters.”

“As a result, the POP_pre_ rapidly increased, doubling doubling from 25 million heads in 1990 to 52 million in 2014 (Fig. 3e).”

should read:

“As a result, the POP_pre_ rapidly increased, doubling from 25 million heads in 1990 to 52 million in 2014 (Fig. 3e).”

